# Experiences of the Patients with Behcet’s Syndrom from Adherence to Treatment: A Qualitative Content Analysis

**DOI:** 10.30476/ijcbnm.2021.89726.1640

**Published:** 2021-10

**Authors:** Narjes Khaton Taheri, Camellia Torabizadeh, Elham Aflaki, Mehdi Mohammadi, Zahra Khademian

**Affiliations:** 1 Student Research Committee, Department of Nursing, School of Nursing and Midwifery, Shiraz University of Medical Sciences, Shiraz, Iran; 2 Community Based Psychiatric Care Research Center, Shiraz University of Medical Sciences, Shiraz, Iran; 3 Autoimmune Diseases Research Center, Shiraz University of Medical Sciences, Shiraz, Iran; 4 Department of Administration and Educational Planning, School of Education Sciences and Psychology, Shiraz University, Shiraz, Iran; 5 Department of Nursing, School of Nursing and Midwifery, Shiraz University of Medical Sciences, Shiraz, Iran

**Keywords:** Adherence to treatment, Behcet’s syndrome, Chronic disease, Qualitative research

## Abstract

**Background:**

Adherence to treatment plays an important role in the effectiveness of treatment in patients with Behcet’s Syndrome (BS). An in-depth understanding of the experiences of patients with BS will help to improve the patients’ management. The present qualitative study aimed to explore the experiences of patients with BS from adherence to treatment.

**Methods:**

This qualitative study was conducted during February-September 2019 at Shahid Motahari Behcet’s Clinic in Shiraz, Iran. Data were collected through eight unstructured observations and 22 individual in-depth semi-structured interviews with 20 participants (15 patients with BS, three family members, and two rheumatologists). Data analysis was performed simultaneously with data collection, using the conventional content analysis as proposed by Graneheim and Lundman. Data were managed using MAXQDA 10 software.

**Results:**

Data analysis resulted in 21 sub-categories, seven categories, and three themes. The themes included barriers to treatment adherence (inability to cope with treatment and challenges in access to health care), facilitators of treatment adherence (incentives, receiving support, and striving to adapt to illness), and missing aspects of the treatment program (inadequate patient education and the neglect of lifestyle modification).

**Conclusion:**

The barriers to and facilitators of treatment adherence as well as the missing aspects of the treatment program of patients with BS were identified. Adherence to treatment is not only determined by the patients, but also affected by the support system and relies upon the existing context and the nature of the treatment plan. These parameters should be considered during patient management to effectively promote treatment adherence.

## INTRODUCTION

Behcet’s Syndrome (BS) is a serious multisystem auto-inflammatory disorder which is prevalent worldwide. However, it is more common along the Silk Road with the highest prevalence in Turkey, Iran, and Japan. ^1^ The prevalence rate of BS in Iran is 80 per 100,000 people. ^2^ BS occurs in repeated cycles of attacks and remissions, but its frequency and duration are unpredictable and do not follow a specific pattern. ^3^ The clinical manifestations of the disease include oral aphthous ulcers; genital ulcers; skin lesions; and ocular, vascular, articular, gastrointestinal, urogenital, pulmonary, and neurologic involvement. ^4^ The main treatment modality is an immediate suppression of the inflammation during acute attacks with corticosteroids to prevent damage and immunosuppressive drugs on a need basis to prevent relapses. ^1^ There are other treatment alternatives; however, their effectiveness and success heavily depend on the patient’s adherence to treatment. ^5^

Adherence to medication plays an important role in the effective treatment of chronic diseases. Conversely, non-compliance may reduce the effectiveness of treatment, increase morbidity and mortality, and increase the healthcare costs. ^6^ Adherence to treatment is defined as the extent of overlap between the behaviour of a patient (taking medication, diet, change of old habits, and regular visits to clinics) and medical recommendations. ^7^ Adherence denotes a proactive attitude of a patient and is indicative of collaboration and interaction patterns between the patient and treatment team. The success of a treatment program depends entirely on the extent to which these parties actively work together as a team. ^8^ A previous study reported that the adherence rate to the prescribed medication regimen among patients with rheumatoid arthritis was 30%. ^7^ Another study in Iran found that only 49.3% of patients with BS adhered to their treatment, so that only 59.9% of them followed the correct time and dose of the medications, and 69.3% of them had regular medical visits on time. ^6^

A review study reported that non-adherence to treatment in patients with chronic diseases was in part due to treatment-related factors, such as the complexity of the treatment, potential side effects, and costs. ^9^ A qualitative study in patients with hypertension identified four main reasons for non-adherence to the prescribed medication, namely day-to-day problems in life, treatment process incompatible with the patient’s behaviour, irregular medication intake, and incorrect medical advice from family members. ^10^

The nature of BS is complex and poor adherence to treatment is correlated with poor patient’s outcome. ^1 , 11^ However, few studies have been performed on treatment adherence in these patients. ^6^ In order to expand the body of knowledge in this field, it is necessary to conduct further studies on this concept among patients with BS. An in-depth understanding of the experiences of the patients will help experienced nurses to identify the determinants of treatment adherence and use them as the basis for effective counselling and patient care. ^10^ Such an understanding can be achieved through qualitative studies. Some available qualitative studies have been conducted on the patients’ experience of adherence to medications in relation to health conditions such as hypertension and diabetes. ^8 , 10^ However, very few have addressed the experiences of patients with BS. ^12^ Therefore, the objective of the present qualitative study was to explore the experiences of patients with BS from adherence to treatment.

## MATERIALS AND METHODS

The present qualitative study, using conventional content analysis, was conducted during February-September 2019 at Shahid Motahari Behcet’s Clinic, affiliated to Shiraz University of Medical Sciences, Shiraz, Iran. The clinic is the main referral centre in Southern Iran. The target population consisted of adult patients diagnosed with BS, their family members, and the rheumatologists involved. The purposive sampling method was used to recruit participants with maximum variation in terms of social background, disease characteristics and treatment, and duration of illness. The inclusion criteria for the patients were 18 years of age and over, a minimum of six months passed from diagnosis of BS, the ability to provide information, and fluency in Persian. The exclusion criteria were having a debilitating disease and unwillingness to participate. The sampling continued until the participants could not provide new information and no new substantive code or category could be acquired (data saturation). Accordingly, a total of 15 patients were recruited. In addition, three family members of some of the participants and two attending rheumatologists were requested to participate in the study. 

Data were collected through 22 individual in-depth semi-structured interviews with 20 participants and eight unstructured observations. The face-to-face interviews were held in a private room in the BS clinic by the first author and lasted 45-60 minutes. Two participants were interviewed a second time to clarify ambiguities. The interview started with a general question, “Please describe your treatment-related experiences?” followed by a series of probing questions to fully explore their experiences with adherence to treatment and to provide an opportunity to clarify the statements. The interviews were audio-recorded. At the end of each interview, audio-recordings were transcribed verbatim.

Concurrent with interviews, 8 unstructured observations were conducted by the first author to complement and enrich the data. With prior permission, the observations were recorded in the form of field notes that included a description of the physical environment and the body language of the patients and their companions. In addition, the behaviour of the health care team and patient-peer interaction was also recorded as field notes. 

The data were analyzed using conventional content analysis, as proposed by Graneheim and Lundman. ^13^ At the end of each interview and observation, audio- recordings and field notes were transcribed verbatim. After a thorough study of the transcript, the initial codes were identified. Extracted codes were then classified in terms of similarities and differences, based on the subcategories which were defined. These were then reviewed, compared, and grouped to determine the main categories. Similar categories were merged and themes were extracted. MAXQDA 10.0 software was used for data organization.

Data trustworthiness was assessed using the four criteria proposed by Guba and Lincoln, namely credibility, dependability, confirmability, and transferability. ^14^ To ensure credibility, we carried out prolonged engagement with the data, peer debriefing, and member checking by five patients. Dependability of the data was evaluated by peer debriefing. To ensure the conformability, we maintained an audit trail, and each stage of the research was carefully noted and documented for future reference. For transferability, all documentation was made available to other researchers. 

### Ethical Considerations

The study was approved by the Ethics Committee (code IR.SUMS.REC.1398.034) and the Clinical Research Administration of Shiraz University of Medical Sciences, Shiraz, Iran. The participants were informed about the objectives of the research and optional withdrawal at any stage of the study was permitted. Furthermore, the confidentiality of any disclosed information was guaranteed. Written informed consent was obtained from all the participants for recording the interviews and participation in the study.

## RESULTS

The mean age of the participants was 45.4±10.58 years. The mean duration of BS illness in patients (n=15) was 10.5±5.01 years (range: 1 to 18 years). Demographic characteristics of the participants are presented in Table 1.

**Table 1:** Demographic characteristics of the participants

Participant	Sex	Age (years)	Marital status	Job	Education Level	Illness duration (years)/Relation to the patient
P1	Female	33	Married	Housewife	Diploma	12
P2	Female	61	Married	Housewife	Diploma	17
P3	Female	44	Single	Housewife	Diploma	9
P4	Female	47	Married	Housewife	Diploma	7
P5	Male	49	Married	Self-employed	Diploma	12
P6	Female	47	Married	Housewife	Diploma	13
P7	Female	66	Married	Retired	Bachelor’s degree	18
P8	Female	49	Married	Housewife	Diploma	1
P9	Female	36	Single	Public employee	Bachelor’s degree	15
P10	Male	56	Married	Retired	Associate degree	12
P11	Female	54	Married	Housewife	Diploma	1.5
P12	Male	40	Married	Public employee	Bachelor’s degree	8
P13	Male	42	Married	Farmer	Illiterate	10
P14	Female	53	Married	Housewife	High school	12
P15	Male	33	Single	Public employee	Bachelor’s degree	2
P16	Female	45	Married	Housewife	Diploma	Family member (spouse)
P17	Female	28	Married	Housewife	Bachelor’s degree	Family member (daughter)
P18	Male	38	Single	Public employee	Bachelor’s degree	Family member (son)
P19	Male	45	Married	Rheumatologist	Medical degree	Physician (junior)
P20	Female	53	Single	Rheumatologist	Medical degree	Physician (senior)

Analysis of data resulted in 1,654 primary codes, 21 sub-categories, seven categories, and three themes (Table 2). The themes were “Barriers to treatment adherence”, “Facilitators of treatment adherence”, and “Missing aspects of the treatment program”.

**Table 2:** The extracted sub-categories, categories, and themes from the interviews and observations

Sub-categories	Categories	Themes
Denial of illness	Inability to cope with treatment	Barriers to treatment adherence
Disease concealment		
Discontinuation of medication due to adverse effects		
Financial burden of treatment	Challenges in access to health care	
Difficulties in scheduling appointments		
Transportation barriers		
Hope for recovery	Incentives	Facilitators of treatment adherence
Combining faith with action		
Fear of the consequences of not pursuing treatment		
Family support	Receiving support	
Peer support		
Interaction with and medical guidance from the treatment team		
Acceptance of illness	Striving to adapt to illness	
Having medication reminder systems to overcome forgetfulness		
Periodic health visits		
Doubts on treatment effectiveness	Inadequate patient education	Missing aspects of the treatment program
Discontinuation of treatment after remission		
Inadequate treatment awareness		
Neglect of physical activity	Neglect of lifestyle modification	
Lack of a balanced diet		
Inadequate management of psychological distress		

### 1. Barriers to Treatment Adherence

Patients participating in the study reported some barriers to adherence to treatment. These barriers hindered them from seeking treatment or discouraged them from continuing the treatment. The categories extracted from this theme were *“inability to cope with treatment” and “challenges in access to health care”*.

#### 1.a. Inability to Cope with Treatment

Based on the patients’ experiences, we found that upon the onset of BS, the patients adopted various approaches to avoid visiting a physician and seek treatment. To cope with the disease, some took refuge in denial or concealment of the disease. Two patients stated:

*“I do not suffer from BS. It is a minor aphtha which does not require specific medication or treatment.”* (P8)

*“There were times that I could not take my medication as I was with my friends. I really did not want them to know that I suffered from BS.”* (P9)

Most participants had experienced medication side effects such as obesity, diabetes, high blood pressure, nausea, vomiting, and stomach pain. The side effects of corticosteroids, which suppress inflammation and immunity, were the main reason for discontinuation of the treatment. The effects were to the extent that some patients arbitrarily adjusted the dosage without prior consultation with a physician. Some participants perceived the side effects even harder to bear than the illness itself. Two participants stated:

*“At times, I feel it is best to stop taking the medication. I rather cope with the symptoms of aphthous ulcers than become diabetic or end up with osteoporosis.”* (P10)

*“Some patients who need to take two or more prednisolone pills a day reduce their pills to one without their doctor’s permission to experience fewer side effects.”* (P20)

#### 1.b. Challenges in Access to Health Care

The associated sub-categories of access to health care were the financial burden of treatment, difficulties in scheduling appointments, and transportation barriers. Among these, the treatment cost was the main challenge to patients who suffered from chronic BS. A participant stated:

*“I cannot afford the costs of physician visits, tests, or medications. There were been times that I was unable to purchase medication, which in turn worsened my health condition.”* (P4)

Some of the participants expressed their dissatisfaction with issues related to scheduling an appointment as well as transportation to/from health centres. Typical examples were overcrowded clinics that hindered their follow-up care, difficulties in making an urgent appointment, and routine cancellation of an appointment in case of late arrival. A patient stated:

*“Since I live outside Shiraz, I need to arrive a day earlier to personally make an appointment. Even worse, it is likely that I arrive late and miss my appointment because of the travel distance or the fact that I do not know my way in the city.”* (P9)

### 2. Facilitators of Treatment Adherence

Facilitators encouraged the patients to follow treatment plans and helped them along the path of adherence. Based on the experiences of the patients, incentives such as hope, faith and fear of consequences of non-adherence, in addition to receiving support from family members, peers and healthcare providers, and striving to adapt to illness were facilitators of treatment adherence. 

#### 2.a. Incentives

The patients stated that a slight improvement in the illness made them hopeful about full recovery. The hope of being relieved of the excruciating pain due to aphthae and the signs and symptoms of their illness motivated them to rigorously continue with the treatment. A participant stated:

*“When I experienced the beneficial effect of medications and pain relief, I gained hope and pursued the recommended treatment.”* (P6)

An important topic that motivated the patients to adhere to treatment was faith and trust in the supernatural power of God. It gave them the feeling of being in control of the illness; therefore, they wanted to pursue treatment. A participant stated:

*“I gain a sense of empowerment when I read the Qur’an or perform daily prayers. Faith in God is an important part of the treatment. Besides doing my utmost to help myself and follow my physician’s recommendations, I trust God for healing.”* (P12)

The participants also stated that the fear of the consequences of not pursuing treatment was an incentive to pursue the treatment regimen. Some patients stated that fear forced them to strictly follow the recommended usage and dosage. They were afraid of side effects such as worsening of the symptoms, exacerbating eye disorders, blindness, and even death. A participant stated:

*“I strictly follow the recommended usage and dosage of my medications. Otherwise, it results in inflammation of the eye and I am afraid of going blind.”* (P5)

#### 2.b. Receiving Support

All participants emphasized the importance of receiving support from family members, peers, and the treatment team in facilitating adherence to treatment. Based on their experiences, the patients indicated that receiving support provided by their family encouraged them to adhere to medications, periodic visits to physicians, and improve self-care. A participant stated: 

*“My husband was very helpful to me. He supported me. He accompanied me on visits. ... When I first had this disease, … he always told me to take my medicine on time. Sometimes he even paid more attention to my appointments. Early in my illness, my baby was small and I was sick. He helped me a lot in taking care of the baby. God bless him.”* (P6)

At times, patients sought receiving support from other patients with comparable illnesses to find the courage to continue with the treatment. In addition, learning adaptive coping strategies from peers motivated them to keep striving for greater adherence to treatment. A participant stated:

*“It is a great feeling to see how patients help each other, share their experiences, and learn from each other. Patients are encouraged to further pursue treatment through the support provided by a treated patient.”* (P18)

The participants emphasized the need for receiving support and effective human interaction with the treatment team as an incentive to continue the path of treatment adherence. Most participants stated that a rational approach in combination with special concern for their illness by the treatment team was an effective factor for their adherence. A participant stated:

*“My physician warned me about discontinuing the treatment and the adverse effect it would have on my eyes. He gave me a clear choice between adherence to treatment and vision loss. The warning was clear enough for me to take the treatment very seriously.”* (P9)

#### 2.c. Striving to Adapt to Illness

This category included the sub-categories of acceptance of illness, having medication reminder systems to overcome forgetfulness, and periodic health visits. The main priority of all participants was to keep BS under control during the recovery period. Some participants managed to deal with the illness by accepting the condition and the limitations caused by the illness. In addition, they sustained adherence through illness acceptance and coping strategies. A participant stated:

*“I was not the type of person to believe in medications and treatment. After becoming ill, I learned that I had no option but to accept the condition, confront it, and seek treatment.”* (P3)

The main treatment regimen for patients with BS involves a regular consumption of medication. Some patients stated that at times they forgot to take the medicine due to various reasons, such as negligence, everyday occupation, and fatigue. A participant stated: 

*“Besides my household chores, I work full-time. By the end of the day, I am dead tired and simply forget to take my medicine.”* (P12)

To overcome forgetfulness, some participants resorted to certain measures such as the use of a weekly pillbox organizer, frequent self-reminder, easy access to medicines, use of written medicine schedules, or reminders from family or friends. A participant stated:

*“I stick a note on the wall of the room or other places that are visible, such as on the refrigerator door. I write on it: Hello dear, you will get better by taking your medicine. Then, I write at this hour you should take your medicine, do not forget.”* (P11)

Some participants were well-aware of the importance of periodic health visits. Such visits contributed to the control of the disease and immediate identification of any abnormalities. A participant stated:

*“I have regular medical checkups and visit gynecologists, ophthalmologists, and others. This helps me to detect any abnormalities on time to avoid having to resort to higher dosages of the medicine.”* (P3)

### 3. Missing Aspects of the Treatment Program

The data indicated that non-adherence to treatment was not only due to the patients; shortcomings in the treatment program also played a role. We noted that certain elements in the BS treatment and care program were neglected. The extracted categories associated with this issue were inadequate patient education and the neglect of lifestyle modification.

#### 3.a. Inadequate Patient Education

Some of our patients wrongly believed that the prescribed medications neither helped them toward recovery nor improved their quality of life. Medications were taken at random, mainly when symptoms worsened. A participant stated:

*“I reached the conclusion that it was no longer needed to take the prescribed medicines because I felt no effect. I saw no point in taking them.”* (P13)

Some patients discontinued the treatment due to a knowledge deficit on the importance of the medications or when the symptoms were mild. They incorrectly perceived the use of medicine to be intermittent and discontinued the medication after remission. A participant stated: 

*“For a while, I found out that my mother did not take her medicine. She did not even visit the physician every three months. I told her,” Mom, why didn’t you go to the doctor? Do you not take medicine? She said: I have no symptoms. I take my medicine every time I get aphthae. I definitely should not always take medicine.”* (P18)

In some cases, the patients were not adequately aware of the disease, medication, and treatment process. In addition, they had a pessimistic view of BS and its management. In this regard, a physician said:

*“Sometimes, we see that the patients say: Doctor! what is the need for so many drugs? They do not have accurate information about BS and do not consider the disease serious. They may even use traditional and herbal medicines aphthae, and as their symptoms improve, they abandon their treatment.”* (P20)

The patients’ lack of awareness of their disease and treatment could have been the result of the neglect of the patients’ needs for education and guidance from the treatment team. On the other hand, some patients were aware that knowledge of BS health-related information was important for recovery. Therefore, they proactively sought information from a variety of sources such as the treatment team, other patients with BS, and literature. However, such personal actions were not an ideal substitute for proper and systematic training and education. A participant stated:

*“Physicians do not provide guidance on the consequences of irregular usage or discontinuation of the medications. They either do not have the time or cannot be bothered to do so. The least they can do is to train the nurses and give them the responsibility of educating the patients.”* (P8)

#### 3.b. Neglect of Lifestyle Modification

Some participants incorrectly assumed that the treatment program only comprised of taking the medicines and attending periodic health examinations without having to consider lifestyle modifications. As a direct result, some participants did not follow dietary requirements to prevent oral aphthous ulcers or side effects of corticosteroids. A participant stated:

*“My personal experience is that eating walnuts, honey, or eggplant triggers aphthous ulcers. I had to find this out by myself because my physician never discussed nor asked about dietary requirements.”* (P11)

Some patients neglected physical activities despite its positive effect on BS symptoms and the reduction of the side effects of corticosteroids. Lack of time, negligence or disinterest was the cause of negligence although some could not exercise due to physical limitations. A participant stated:

*“My physician never told me about the benefits of exercise on my illness. The only physical activity I have is walking in the neighbourhood if I feel up to it.”* (P10)

Some participants suffered from inadequate management of psychological distress, which, in turn, made it more difficult to adhere to the treatment. Typically, they showed tension, anger, depression, mood swings, and boredom. A participant stated: 

*“At times, I do not take my medications due to stress, boredom, and internal family conflicts.”* (P3)

## DISCUSSION

In the present study, an attempt was made to explain the experiences of patients with BS from adherence to treatment by in-depth interviews and observations. The results showed three themes of barriers to treatment adherence, facilitators of treatment adherence, and missing aspects of the treatment program. 

 In the current study, the factors which prevented the patients’ adherence were inability to cope with treatment and challenges in access to health care. Other studies also identified inconsistent psychological responses in patients with chronic diseases as one of the most common reasons for incompatibility. ^15 , 16^ A previous study reported that denial of illness was often accompanied by stubborn behaviours such as avoidance of treatment follow-up and refusal to take the prescribed medications. ^17^ In addition, the social stigma associated with a disease forced the patients to conceal their illness from others, even to the extent of hiding the use of prescribed medications from family members. ^18^

In the current study, the adverse effect of medications was another barrier to adherence, causing fear, increased risk of treatment discontinuation, and irregular usage of the prescribed medications. Similarly, a previous qualitative study indicated that the patients’ perception of the adverse effects outweighed the benefits of medications. ^19^ Further challenges associated with access to health care were the financial burden of treatment, difficulties in scheduling appointments, and transportation barriers. Other studies also indicated that treatment costs, particularly in patients with chronic diseases, adversely affected adherence to medication. ^20 , 21^ A previous study conducted in North Carolina reported that a 10% increase in treatment adherence resulted in a reduction of treatment costs by up to 25%. ^22^ Therefore, financial support to low-income patients through governmental or charity organizations would have a positive effect on their adherence to treatment. 

BS is a type of illness that requires continuous care. ^1^ The current findings showed that access to health care was overshadowed by issues related to the scheduling of appointments, and problems related to the costs of treatment and transportation to the clinic. Among our patients, over 50% lived outside Shiraz city and had to travel a long distance to reach the BS Clinic. Other studies also reported similar issues for patients. ^23 - 25^ We strongly recommend resolving these issues and supporting BS patients to better adhere to treatment. 

Facilitators of treatment adherence were incentives, receiving support, and striving to adapt to the illness. The results showed that hope for recovery, faith, and fear of the consequences of not pursuing treatment encouraged adherence to treatment. Previous studies have shown that hope in patients with rheumatoid arthritis and diabetes was directly associated with improved adherence to treatment. ^26 , 27^ In the present study, cultural tendencies and religious beliefs of the participants were important sources of hope enabling them to accept and adapt to the illness. Previous studies have also reported that some patients believe their illness is God’s will and the supernatural power of God has control over it. ^28 , 29^

We found that the fear of BS complications (blindness, death) was the trigger for taking the illness seriously and better adhering to the treatment. Educating such patients on the potential complications of BS has been recommended. ^23 , 25^ Our data also showed that receiving support was an essential factor in treatment adherence so much that adherence without such support seemed unimaginable. This was in line with previous studies in which receiving support from family members played an important role in the treatment and self-care of patients with chronic diseases and had a positive effect on treatment adherence. ^18 , 22^

Several studies have shown that treatment adherence is positively affected by an effective interaction between the treatment team and the patient, another essential element of support. ^12 , 27 , 30^ A study in Singapore reported that adherence to treatment was directly related to the patients’ faith in physician recommendations, promoting acceptance of the proposed treatment regimen. ^25^ The current findings indicated that patients are motivated to adhere to the proposed treatment program through counselling, interaction with the treatment team, and guidance for coping with BS. In addition, peer support contributed to treatment adherence by sharing their lived experiences of the illness. Various studies have highlighted the important role of peer support in improving physical conditions and the empowerment of patients with chronic diseases. ^12 , 31 , 32^ We recommend creating and facilitating peer support groups to help the patients with BS.

The results of the present study showed that patients tried to adapt to their condition through acceptance, periodic health visits to facilitate adherence to treatment, and by putting reminders in place to overcome forgetfulness. It has been shown that the higher the degree of disease acceptance, the less severe the side effects and negative feelings associated with the disease, and the greater the patient’s participation in the treatment. ^33^ In our study, due to a variety of personal and medical problems, the patients paid less attention to the treatment regimen and often forgot to take their medications. Other studies have also associated forgetfulness with non-adherence to treatment. ^10 , 25 , 29^ To overcome forgetfulness, they resorted to various medication reminder systems. An overview on systematic reviews revealed that electronic reminders were effective strategies to improve the patients’ adherence to treatment and medication. ^34^ Overall, both incentives and the support systems need to be strengthened to motivate the patients to adhere to the treatment program and adapt to their illness. 

We found that not only non-adherence to treatment was related to the patients, but shortcomings in the treatment program also negatively affected the condition. The lack of a specific plan for lifestyle adaptation and omission of effective health education exacerbated non-adherence to treatment. A number of quantitative and qualitative studies have shown the importance of sufficient knowledge of and a positive attitude towards the effectiveness of medications. ^35 , 36^ Insufficient knowledge is shown to contribute to ignoring medical recommendations, doubts about treatment effectiveness, and the discontinuation of medications. ^29 , 35^ Mental conditions such as anger, depression, and boredom also contribute to non-adherence. ^20 , 37^ Giving advice on the effect of medications not only reduces the patients’ anxiety, but also eliminates any misconceptions. ^38^ Furthermore, education and training could facilitate the patients’ adjustment to their lifestyle. Specific measures are required to ensure optimal adherence to treatment by patients. ^39^ In the present study, the absence of educating the patients by the treatment team and the lack of comprehensive care by nurses was evident. Moreover, there was complete neglect of the important role that nurses could play in patient education, despite the approved positive effect that dedicated education could have on the quality of life of patients with chronic diseases. ^40^

 As the main strength of this study, we conducted one of the very few qualitative studies that evaluated adherence to treatment in patients with BS. In the present study, we included the experiences of all parties involved, not only from the patients but from their family members and physicians as well. The participants were chosen based on the principle of maximum variation, which added to the diversity of experiences. The main limitation of the study was that we used the qualitative content analysis method to explore the experiences of patients with BS from adherence to treatment. Using a design such as phenomenology will help to better understand the patients’ lived experiences of this concept. 

## CONCLUSION

The current findings revealed that adherence to treatment is not only determined by the patient’s adjustment with treatment, motivation, and access to services, but is also affected by the support system and the nature and comprehensiveness of the treatment plan. Health care professionals can utilize our findings to improve the patients’ follow up and their treatment adherence, in addition to facilitation of fair access to treatment facilities. Further studies can use these findings as a basis for developing comprehensive interventions to improve adherence to treatment among patients with BS. 
